# Nutritional Management of Oncological Symptoms: A Comprehensive Review

**DOI:** 10.3390/nu15245068

**Published:** 2023-12-11

**Authors:** Mattia Garutti, Claudia Noto, Brenno Pastò, Linda Cucciniello, Massimiliano Alajmo, Amanda Casirati, Paolo Pedrazzoli, Riccardo Caccialanza, Fabio Puglisi

**Affiliations:** 1 CRO Aviano, National Cancer Institute, IRCCS, 33081 Aviano, Italy; 2 Department of Medicine, University of Udine, 33100 Udine, Italy; 3 Le Calandre Restaurant, Alajmo Group, 35030 Sarmeola, Italy; 4 Clinical Nutrition and Dietetics Unit, Fondazione IRCCS Policlinico San Matteo, 27100 Pavia, Italy; 5 Department of Internal Medicine and Medical Therapy, University of Pavia, 27100 Pavia, Italy; 6 Medical Oncology Unit, Fondazione IRCCS Policlinico San Matteo, 27100 Pavia, Italy

**Keywords:** cancer, nutrition, stomatitis, xerostomia, diarrhea, nausea, vomiting, dysphagia, anorexia, cachexia

## Abstract

Throughout their experience of illness and during the course of treatment, a substantial proportion of cancer patients are prone to develop nutritional and/or metabolic disturbances. Additionally, cancer patients often encounter long-term side effects from therapies, which may lead to impaired digestion, nutrient absorption or bowel motility. Therefore, the preservation and maintenance of an optimal and balanced nutritional status are pivotal to achieving a better prognosis, increasing the tolerance and adherence to cancer therapies and improving the overall quality of life. In this context, personalized nutritional programs are essential for addressing conditions predisposing to weight loss, feeding difficulties, digestion problems and intestinal irregularity, with the goal of promoting adequate nutrient absorption and minimizing the detrimental effects of treatment regimens. The focus of this research is to examine the most common clinical conditions and metabolic changes that cancer patients may experience, including stomatitis, xerostomia, diarrhea, nausea, vomiting, dysphagia, sub-occlusion, dysgeusia, dysosmia, anorexia, and cachexia. Furthermore, we present a pragmatic example of a multidisciplinary workflow that incorporates customized recipes tailored to individual clinical scenarios, all while maintaining the hedonic value of the meals.

## 1. Introduction

In cancer patients, advances in oncological treatments have significantly improved survival rate [1,2].

However, anticancer therapies can lead to nutritional and/or metabolic alterations, and an insufficient consideration of nutritional status during treatment carries significant implications for patients’ quality of life and their capacity to adhere to different available treatments, thereby adversely affecting prognosis [3].

Furthermore, even when a state of malnutrition is detected, corrective measures are often not adequately implemented [1].

In this context, preserving an optimal and well-balanced nutritional profile enhances the tolerance to cancer therapies, enabling the preservation of a good quality of life (QoL) [4,5].

Indeed, precise nutritional guidance is required depending on the specific disease, treatment, and the patient’s clinical conditions.

Personalized nutritional programs are essential in all those conditions predisposing to weight loss and difficulties in feeding, digestion, and intestinal regularity, so as to promote adequate nutrient absorption and minimize the detrimental effects of treatment regimens.

From the initial assessment and throughout the later stages of the disease, it is imperative to evaluate the dietary intake of cancer patients to promptly detect any nutritional deficiencies. Neglecting these deficiencies can lead to a decline in their nutritional status and have a substantial impact on the effectiveness of cancer treatments, ultimately diminishing the patient’s chances of survival [1,6]. In this context, through a literature review, we present an overview of the prevalent clinical conditions and metabolic changes observed in cancer patients. Our aim is to offer a concise reference guide encompassing primary nutritional strategies, including both pharmacological and non-pharmacological interventions, designed to mitigate and address the symptoms experienced by cancer patients, thereby optimizing their overall outcomes. In addition, we provide a practical illustration of a multidisciplinary workflow for incorporating customized recipes tailored to specific clinical scenarios. 

## 2. Material and Methods

**Figure 1:**
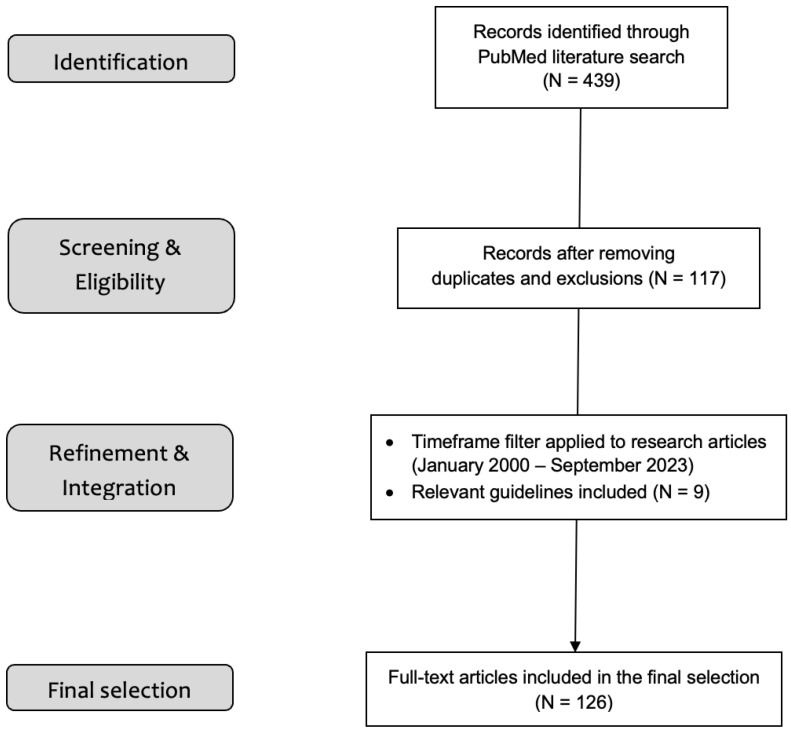
Flow diagram of the literature search process.

We first performed a literature screening to find relevant titles and abstracts about nutrition and clinical conditions in cancer patients (Figure 1). After that, a more extensive PubMed literature search was conducted to identify key clinical trial reports and review articles covering the most prevalent side effects of common anticancer treatments. The search strategy comprised the following keywords: “xerostomia” (OR “stomatitis” OR “mucositis” OR “oral mucositis” OR “diarrhea” OR “constipation” OR “nausea” OR “vomiting” OR “dysphagia” OR “sub-occlusion” OR “dysgeusia” OR “dysosmia” OR “anorexia” OR “cachexia”) AND (“nutritional status” OR “nutrition”) AND (“cancer patients” OR “cancer” OR “neoplasia” OR “tumor”). Relevant narrative and systematic reviews, as well as meta-analyses and reports of small retrospective and prospective clinical trials, were included in order to provide a comprehensive description of a specific clinical problem. Such descriptions would cover a general definition of the issue, epidemiological data, the most common presenting symptoms, and pharmacological and non-pharmacological treatment options. Initially, neither formal inclusion and exclusion criteria nor time filters were applied. Later, the resulting first draft of the literature search was refined using the CrossRef system, excluding possible duplicated, corrected or retracted articles, and the timeframe was limited to research papers published between January 2000 and September 2023. Relevant nutritional recommendations, as well as guidelines published by the Multinational Association of Supportive Care in Cancer (MASCC) and by major oncology societies, were also included in the final selection, which was made by a panel of four experienced medical oncologists; only full-text English articles were considered.

After presenting an update on the above-mentioned side effects of anticancer therapies in the form of a narrative review, we developed a draft of a conceptualized multidisciplinary platform, consisting of a 5-step workflow, to test specific food recipes for three selected clinical scenarios. The involved team was composed of six medical oncologists, two nutritionists and a three-Michelin-star chef. Further details on such nutritional workflows are provided below in a specific section of the present work.

### 2.1. Xerostomia

Xerostomia refers to a subjective feeling of oral dryness often associated with a decreased salivary secretion [7]. This condition may affect up to 40% of the global population, with a higher prevalence among older adults and postmenopausal women [8,9,10].

The primary predisposing and contributing factors encompass autoimmune diseases (such as Sjögren syndrome and rheumatoid arthritis), endocrine disorders (uncontrolled diabetes mellitus, and thyroid diseases) and viral infections (HIV, HTLV-1, and HCV) [7,11].

In addition, in cancer patients undergoing radiation therapy, xerostomia stands out as the most frequently reported oral symptom [12,13], particularly in patients with head and neck (H&N) cancer. For these patients, salivary gland hypofunction depends on the cumulative radiation dose administered and the volume subjected to irradiation, leading to a potentially dramatic impact on QoL [14,15,16]. 

Similarly, some chemotherapy agents like anthracyclines, cyclophosphamide, 5-fluorouracil (5-FU) and methotrexate are also able to reduce salivary gland function that is typically less severe and reversible after completion of treatment [17]. Other drugs associated with xerostomia are antidepressants, beta-2-adrenergic agonists, inhaled steroids, and benzodiazepine derivatives (diazepam, lorazepam) [7,8]. 

Clinical manifestations of xerostomia mainly affect the oral cavity and the upper part of the digestive tube and include difficulties in food ingestion, swallowing and even speaking in severe cases, burning mouth and/or tongue, fissures in lips and mouth mucosa, and susceptibility to caries [7,8]. 

Therefore, symptomatic treatment to mitigate the abovementioned adverse effects of salivary gland hypofunction and xerostomia is important. 

Pharmacological agents stimulating muscarinic receptors, like pilocarpine, bethanecol, and cevimeline are reported to be effective and may be offered after radiotherapy, even though the improvement is only transitory [18,19,20]. Saliva stimulants (e.g., sugar-free, non-acidic chewing gums), topical lubricants and saliva substitutes are especially recommended when it is still possible to increase saliva flow rate to relieve symptoms associated to xerostomia, although the long-term benefits are still uncertain [18,19,21]. Acupuncture has also been tested both during and after radiation therapy in H&N cancer patients, and while there is little evidence that this intervention is able to increase saliva flow rate, some patients have experienced an improvement in xerostomia-associated symptoms [18,19,22].

While there is no standard, highly effective treatment for the prevention and management of xerostomia, it is strongly advisable to maintain proper oral hygiene through regular teeth cleaning. This practice serves to mitigate the risk of potential infections, such as oral candidiasis, and to uphold the highest possible QoL [19]. Nutritional counselling is another important resource to provide to these patients. Based on everyday life and insights from previously published works [23], some fundamental suggestions can be offered to patients, focusing on introducing different flavors such as marinating meat, poultry or fish or cooking them in naturally sweet juices; increasing the use of spice, herbs and seasonings; preferring fresh vegetables (also in combination with tasty dressings) or fruit smoothies without neglecting the importance of regular and small sips of fluids. Moreover, it is preferable to flavor boiled or steamed foods by adding salt. 

Because gastric acid production increases saliva secretion, it would not be incorrect to think that sour foods may be beneficial for those patients with partial xerostomia, as opposed to total xerostomia where acidic foods may be avoided [24]. 

Conversely, certain suggestions may be inappropriate. These include ice chips/cubes, which offer only brief and inadequate relief, glycerol solutions that can have a drying effect on the mouth, acidic products like lemon solutions and pineapple chunks, which may intensify oral discomfort and contribute to dental demineralization, sweet and candies known for their high cariogenic potential, and vitamin C and antioxidants, as they may have a potential adverse impacts on cancer-related outcomes [19]. 

As a result, while nutrition certainly plays a role in reducing the development and exacerbation of xerostomia, available data remain limited. Investigating the role of early nutritional counselling and refining possible nutritional recommendations might be interesting fields of research in the near future.

### 2.2. Stomatitis

Stomatitis is a very common toxicity of cancer treatments consisting of inflammatory damage that can arise from oral to anal mucosa. It involves around 30–40% of patients receiving chemotherapy, being more frequent among H&N cancer patients in which it occurs up to 90% of cases [25,26].

Risk factors include exposure to radiation, cytotoxic drugs, or newer oral-targeted agents [27,28,29,30,31]. Younger age may also be a contributing factor, possibly due to a higher mucosa turnover, rendering it more susceptible to cytotoxic drugs. Other factors include pre-existing oral defects and diseases, such as poor hygiene, caries, and periodontal disease, as well as an inadequate nutritional status and differences in the oral microbiome [32,33,34]. Pyrosis can also contribute to the development of stomatitis; in fact, it represents an inflammatory alteration to the mucosa of the upper gastrointestinal (GI) tract, most frequently occurring as a consequence of excessive acid reflux from the stomach to the esophagus or even the laryngopharynx [35]. Some preliminary studies have identified possible associations between chronic gastro-esophageal or laryngeal reflux disease and an increased onset of H&N cancers, particularly within the larynx [36]. Moreover, a higher severity of reflux disease has been described as a potential, significant risk factor for more serious radiation-induced mucositis in patients with stage I-II laryngeal and hypopharyngeal cancers [36].

The main clinical manifestations range from oral mucosa erythema to multiple ulcerations and canker sores causing painful swallowing that can lead to diet modifications or difficulty in food or liquid ingestion [37,38,39].

Therapeutic management of stomatitis relies on the multiple approaches available, a combination of which seems to be required, as no single intervention has so far proven real effectiveness per se [27,40].

Saline and sodium bicarbonate mouthwashes are well-consolidated bland remedies which may help symptom management by cleaning the oral cavity, while benzydamine-based mouthwashes with their anti-inflammatory properties have shown to be effective as a preventive measure in patients undergoing chemotherapy and/or radiant therapy [40,41].

On the other hand, topical morphine 0.2% mouthwashes are suggested to reduce stomatitis-associated pain [40]. Furthermore, oral cryotherapy represents another preventive and easy practice playing a beneficial role on the associated dysgeusia and xerostomia [40,42,43,44].

Many natural remedies have also been considered, but a general lack of high-quality evidence is noted for many of them, such as zinc, vitamin D and E, selenium, folinic acid, propolis, and liquorice [42,45,46]. Conversely, honey has shown consistent positive effects in a combined topical and oral administration and is therefore suggested in H&N patients treated with combined treatment; however, due to its high cariogenic potential, patients should adhere to a strict oral hygiene program [46,47].

Vitamin D levels have been extensively associated with cell proliferation and cancer [48,49]. Currently, there are not enough literature data to sustain vitamin D supplementation for cancer prevention. Indeed, in a randomized clinical trial, 2303 postmenopausal women were randomly given vitamin D and calcium supplementation or placebo. In this trial, no significant reduction in the risk of all-type cancer was observed after 4 years amongst the two treatment groups [50]. Furthermore, in another randomized clinical trial, 25,871 patients were randomized to receive vitamin D and omega-3 supplementation versus placebo and, also in this case, vitamin D supplementation did not result in a reduced incidence of breast, colorectal or prostate cancer or cardiovascular diseases compared to placebo [51]. The role of vitamin D as a therapeutic agent has also been explored in cancer patients [52,53,54,55]. In the AMATERASU randomized clinical trial, patients with early stage esophageal, gastric and colorectal cancer in follow up were randomized to receive vitamin D supplementation versus placebo. No significant difference in terms of relapse-free survival was observed amongst the two treatments [56]. In a post hoc analysis of this trial, serum anti-p53 antibodies were detected in about 1/3 of the patients. Within this subgroup of p53-immunoreactive patients, a significant improvement in relapse-free survival and in overall survival was reported in the vitamin D group, in contrast to the non–p53 immunoreactive subgroup, in which vitamin D supplementation had no survival impact. Therefore, these findings could suggest that vitamin D supplementation may reduce the risk of disease recurrence and mortality within p53-immunoreactive patients [53]. Similarly, in the SUNSHINE phase II clinical trial, 139 patients with metastatic colorectal cancer were randomized to receive treatment with mFOLFOX-bevacizumab with either high-dose or low-dose vitamin D3 supplementation. In this trial, the addition of high-dose vitamin D3 to conventional chemotherapy resulted in a statistically insignificant improvement in median PFS, thus warranting further investigations [55]. 

Importantly, complicated stomatitis may lead to longer hospitalizations and premature treatment discontinuation [38,57]. Proactively predicting, screening, and detecting early signs of lesions is therefore paramount for cancer patients about to begin treatments [40,45,58].

Aiming at a multidisciplinary preventive management to mitigate such potentially detrimental effects, nutritional counselling might also be integrated, even though evidence on its real utility is still missing.

Dietary suggestions should prioritize the following [40]: preferring soft or semisolid, mild-textured foods like pudding, creamed soups, fresh cheese, and mashed potatoes over rough-textured options; softening food by blending, soaking or moistening it to make it easier to consume; opting for cool or room-temperature foods, as well as tart, acidic or salty flavors, such as tomatoes, kiwi, and lemons; limiting the consumption of raw vegetables and carbonated drinks; using straws or similar devices to bypass severe mouth sores when necessary; and providing high-calorie or high-protein supplements in case of insufficient oral intake or cancer cachexia [23,40].

In summary, many options for managing stomatitis are already available to use; nonetheless, more research focusing on prevention plans, the personalization of treatment and the impact of nutritional management will surely be needed soon, given the high frequency of this condition among cancer patients, its potential impact on QoL and the continuous evolution of cancer therapy.

### 2.3. Diarrhea

The term “diarrhea” refers to the output of abnormally liquid stools at least three times in a day and it is a commonly reported adverse events amongst patients receiving systemic antineoplastic therapy including chemotherapy, target therapy and immunotherapy [59,60]. 

Diarrhea represents a potentially life-threatening complication, as it can cause an important loss of body fluids, consequently leading to hypotension, renal damage, electrolytic imbalances, and cardiovascular events [61]. Thus, it is a major adverse effect requiring prompt pharmacological and non-pharmacological interventions. 

For patients experiencing treatment-related diarrhea, a conservative approach, consisting of dietary modifications (also including an appropriate oral hydration) and anti-diarrheal agents (e.g., loperamide or additionally octreotide for more severe loperamide-refractory cases) can be proposed at first [62].

To restore the electrolyte balance and to avoid the risk of dehydration, abundant oral liquids with solutions rich in electrolytes and glucose (e.g., non-coffee drinks, juices and soups) should be proposed, considering that glucose is a major driver of sodium and water reabsorption [63,64]. 

Moreover, in general, patients with diarrhea can benefit from a diet structured around small and fractioned meals rich in soluble fibers (e.g., white rice, barley, oats, sweet potatoes and some fruits like apples and pears without skin), as they can increase stool consistency and delay bowel movements [65]. Soluble fibers dissolve in water, forming a kind of gel that can help absorb water in the stool, making it less liquid. However, it is important for patients with diarrhea to always consult a healthcare professional for personalized guidance, as dietary needs can vary from person to person and depend on the underlying cause of the diarrhea. In some cases, it may be necessary to avoid some of these sources of soluble fibers if they are not well tolerated.

It is important to note that not all fibers are the same. Insoluble fibers (such as whole grains, corn, and fruits with skin) can have the opposite effect, increasing the frequency of bowel movements, and should be avoided as they can accelerate the GI transit and stool frequency [65,66]. Also, foods and beverages with high osmolarity (i.e., salty foods and carbonated beverages), caffeine and theine should be excluded from the diets of patients experiencing diarrhea. This is because they may increase water excretion in the bowel and consequently increase the stool frequency [67]. 

Probiotics supplementation may offer a viable approach to mitigate treatment-related diarrhea by potentially aiding in the restoration of the integrity of gut microbiota. It is important to note that scientific evidence in this regard remains quite limited [68,69]. However, patients with a central venous catheter should exercise caution and refrain from probiotic supplementations that contain *Saccharomyces boulardii* due to the associated risk of fungaemia [70,71]. 

Moreover, different chemotherapeutic agents can damage the bowel mucosa, consequently leading to malabsorption. Lactose malabsorption and intolerance, which can cause diarrhea, are commonly observed in patients receiving chemotherapy containing fluoropyrimidines; such complications are generally reversed as soon as the treatment is interrupted [72]. However, these patients should be advised to follow a lactose-free diet (i.e., reducing the intake of milk and dairy products) throughout the treatment and, especially, if they develop diarrhea. 

Finally, for more severe cases, hospitalization can instead be required for appropriate intravenous hydration, electrolyte correction and monitoring of the renal and cardiovascular functions [62].

### 2.4. Constipation

Constipation stands out as the prevalent syndrome affecting the intestines in cancer patients undergoing anti-cancer treatment. This condition, with multiple contributing factors, is marked by painful, uneasy, and incomplete bowel movements, along with sluggish and irregular peristalsis. These symptoms can adversely impact the quality of life [73]. As a result, addressing and mitigating constipation is an integral aspect of cancer care.

Depending on its etiology, constipation can be linked to abnormalities on colonic functions (primary) or to medication-adverse events, bowel obstruction/compression or metabolic diseases such as diabetes mellitus, hypothyroidism, hypercalcemia (secondary) [74,75]. 

In cancer patients receiving opioid therapy, constipation affects a substantial proportion, ranging from 15% to 90%, primarily as a result of opioids binding to gut receptors that decrease sensitivity to defecation reflexes [76]. On the other hand, antiemetic agents and certain chemotherapy drugs, such as like vinca alkaloids, platinum-based compounds, and taxanes, can also increase the likelihood of constipation due to their neuropathic effects, which results in enteric neuronal loss and subsequently reduces GI transit time [61]. Therefore, it is imperative to approach constipation management on an individual basis, with a crucial emphasis on pinpointing its precise underlying cause to facilitate the selection of the most appropriate treatment strategy. 

Osmotic and stimulant laxatives are the basis of medical treatment constipation. Lactulose, magnesium hydroxide and polyethylene glycol are examples of osmotic laxatives drawing water into the gut and increasing the stool softness. On the other side, senna and cascara (stimulant laxatives) alter electrolyte transport resulting in intraluminal fluids secretion. If there is noclinical benefit, it can be helpful to employ prokinetic and prosecretory agents (i.e., linaclotide, lubiprostone, and prucalopride) [73,77,78,79,80].

Maintaining an adequate diet should be the first strategy to improve the QoL. Sufficient water intake and a high-fiber diet are recommended help to prevent issues like constipation: drinking at least 1.5–2 L of fluids per day and consuming fiber-rich foods can be helpful in avoiding chemical, mechanical and thermal irritation of the GI tract due to acid-forming fermentation and lower intestinal motility [81,82]. If the patient has difficulty with liquid intake, drinking small sips of liquids that are warm or at room temperature and are varied in flavor can make fluid intake more enjoyable and promote hydration [40,83]. Gradually increasing fiber intake can help the patient’s digestive system adapt and can reduce the risk of digestive discomfort such as bloating, gas, and cramping. The daily recommended fiber intake is at least 25 g, with a 3/1 insoluble/soluble fiber ratio, replacing refined cereals with whole grains [40]. Consuming at least 5 servings of fresh fruits and vegetables per day can provide oncology patients with important nutrients and fiber, contributing to their overall health. Conversely, spicy, sour and salty foods should be reduced, as well as extremely hot or extremely cold ones, due to their irritant properties and lower water contents [40,81,82,83]. 

The use of dairy products containing bifidobacteria and lactobacilli can be helpful in normalizing intestinal microflora. Some dairy products containing these probiotics might help restore the balance of intestinal flora, which may be disrupted during oncology treatment. 

It is important to note that the nutritional needs and wellbeing of an oncology patient can vary significantly depending on the type of cancer, the stage of the disease, and ongoing treatments. Therefore, it is essential for an oncology patient to receive personalized medical guidance from an oncologist and a dietitian to address their specific needs.

It is equally important to respond to the urge to eliminate and to maintain a regular schedule for bowel movements, preferably after breakfast. This can help prevent constipation. In addition, assuming a comfortable position, such as a squatting position, during evacuation can help facilitate the process [81]. 

As can be understood from these guidelines, constipation remains an important and complex issue in cancer patient that should be evaluated using a multidisciplinary approach involving medical oncologists, nutritionists, pharmacists and palliative care physicians.

### 2.5. Nausea and Vomiting

Nausea and vomiting (N/V) are two strictly connected biological phenomena that an organism uses to defend itself from pathological conditions and toxic substances. Nausea is defined as a subjective discomfort of the upper abdomen often followed by a forced and rapid emptying (emesis or vomiting) of the GI content [40,84,85].

Among cancer patients, N/V are frequently triggered by various treatments, including chemotherapy (referred to as CINV—chemotherapy-induced nausea and vomiting) or radiation therapy (known as RINV—radiation-induced nausea and vomiting). In addition to chemotherapy and radiation therapy, it is important to note that new therapies, such as antibody–drug conjugates (ADC), can also induce emesis [86,87]. N/V can manifest in different time frames: acutely (within 24 h after treatment), in a delayed manner (more than 24 h later), or even before treatment is administered (anticipatory emesis) [88,89,90]. 

It is reported that over 60% of advanced cancer patients experience N/V at least once during oncological therapies, with a demonstrated detrimental impact on QoL and on the continuity of care [88,91].

The mainstay of acute and delayed emesis prophylaxis is pharmacological management based on the emetogenic potential of therapies as well as other patient factors and contributing causes. Actually, the major guidelines recommend a combination of two or three drugs in high and moderate emetogenic regimens (e.g., a serotonin receptor antagonist + dexamethasone ± a Neurokinin 1 (NK1) receptor antagonist) [90,92,93,94], while a single agent approach (e.g., metoclopramide) is preferred in low emetogenic agents [90,94,95].

As well as optimizing acute and delayed prophylaxis of CINV and RINV, benzodiazepines and various behavioral interventions (e.g., hypnosis, muscle relaxation, systemic desensitization) [88,89] are useful in minimizing anticipatory nausea. Among the other non-pharmacological interventions, acupressure showed slight improvements in nausea and retching experiences reducing the use of rescue antiemetics [96,97]. Similarly, ginger (*Zingiber officinale*) and cannabinoids have shown similar benefits for N/V management, probably owing to their anti-inflammatory, anti-spasmodic and prokinetic effects [98,99]. Also worthy of note for their ability to relieve the severity of nausea and vomiting are chamomile, peppermint, and cardamom [100,101,102]. 

Due to the increased risk of malnutrition in patients experiencing CINV/RINV, dietary interventions should be considered as an early measure to offer to cancer patients. However, it is important to note that no clinical trials have specifically investigated their role so far [103]. Based on the best practice standards and on expert opinions [23,40,103], several suggestions can be made during nutritional counselling for patients experiencing CINV/RINV. These recommendations can broadly be categorized into three different strategies. 

The initial approach revolves around dietary habits, encompassing the consumption of small, frequent meals, the potential incorporation of light snacks between meals, minimizing extended fasting periods, administering antiemetics before meals, and refraining from eating or drinking until vomiting is managed.

The second strategy involves meal composition and focuses on reducing liquid intake during meals; avoiding the consumption of spicy, fatty, salty food and strong flavors; preferring bland foods, soft fruits or vegetables; choosing low-fat protein sources such as chicken, turkey and eggs; limiting foods that can cause gas or bloating such as pulses, cabbage and beans; and drinking clear liquids or teas, either warm or cold.

Finally, the third strategy includes eating in well-ventilated rooms with no food smells.

Interestingly, emerging from the preliminary data of small prospective trials [103,104] is the antiemetic effect of protein-rich meals, which seems to determine a significant trend towards reducing CINV, both alone and in combination with ginger. Although this mechanism is not fully elucidated yet, this may represent a novel nutritional approach to help overcome N/V in cancer patients. 

In conclusion, despite the fact that much progress has been made in the treatment of CINV/RINV, these are still frequently reported by patients and affect tremendously their QoL. It is thus critical that research continues to further understand pathways underlying N/V, so as to develop multimodal approaches, comprising not only pharmacological, but also non-pharmacological and possibly nutritional interventions [40,105].

### 2.6. Dysphagia

The term “dysphagia” is used to indicate the difficulty in the transit of liquids and/or solid foods either from the oral cavity to the pharynx (“oropharyngeal dysphagia”) or through the esophagus (“esophageal dysphagia”) [59]. It is a common complication of different types of cancers (e.g., oral, esophageal, H&N and central nervous system (CNS) cancers), but it can also arise as a consequence of chemoradiation therapy or surgical procedures in these regions [40]. Dysphagia could be caused by a neurological impairment of swallowing (neurologic dysphagia) or by a mechanical obstruction. 

The material that is not swallowed can eventually enter the trachea and the lungs, leading to suffocation, aspiration pneumonia and other pulmonary disorders. Furthermore, dysphagia has been related to reduced caloric intake, weight loss and malnutrition, thus jeopardizing the health status and the survival outcomes of cancer patients [40,59]. Thereafter, it requires timely recognition and management, which can include both chemoradiation and surgery to shrink the tumor, thereby improving swallowing function, along with specific nutritional recommendations. 

In patients presenting with dysphagia for which oral nutrition is contraindicated due to the disease itself or the ongoing treatment causing significant swallowing difficulties, or in cases where the patient has reached an end-stage disease with no chance of improving their dysphagia, enteral nutrition can be considered. Enteral nutrition can be administered through either a nasogastric tube or a percutaneous endoscopic gastrostomy (PEG) [72]. Among these options, PEG is considered a safer and more feasible choice. 

Alternatively, when oral nutrition remains a viable option, it is advisable to recommend nutritional counseling to establish specific dietary guidelines to assist patients in maintaining an adequate daily calorie intake and preventing malnutrition and weight loss. Modifying the texture and viscosity of liquids, as well as semi-solid foods, can help to reduce the risk of aspiration [106]. Patients with dysphagia should be encouraged to consume small, fractionated semi-solid meals and prioritize foods with a firmer texture, such as cooked vegetables. These foods, when chewed, should not break apart in the upper digestive tract but instead form a compact bolus [40]. 

Similarly, to reduce the risk of choking and aspiration, sticky or bulky foods should be avoided. Moreover, liquids should be consumed with care, considering the risk of entrance into the pharynx. Therefore, thick liquids (e.g., yogurts) should be preferred, or fluids can be thickened [40]. Additionally, avoiding mixed textures and promoting the use of fats may facilitate the swallowing of food bolus. However, in cases of mechanical dysphagia, a more liquid form of foods and a reduction of fiber content could improve the swallowing process. 

It is also important to provide patients with guidance on physical therapy and the correct posture to adopt during meals. For instance, exercises aimed at strengthening the jaw muscles and improving chewing and swallowing functions have been shown to be effective in managing dysphagia and increasing nutritional intake [106]. Furthermore, maintaining the proper body posture while eating is fundamental. 

Patients experiencing dysphagia should, therefore, be instructed to always consume their meals when sitting; avoid lying down for a few hours (typically three hours) after eating; consider undergoing speech therapy (logopedic rehabilitation); and consider an appropriate examination of the oral cavity after meals to remove any food residue [40]. These measures can help improve the management of dysphagia and enhance the safety and effectiveness of eating for patients with swallowing difficulties.

### 2.7. Sub-Occlusion

Upper and lower GI sub-occlusion is a typical complication of different types of advanced cancers, such as GI and gynecological cancers. It is a condition that refers to the mechanical or functional obstruction of part of the bowel (the small and/or the large bowel), thus blocking the passage of the food. Impaired food transit related to an upper GI obstruction typically leads to regurgitation and vomit, whilst lower GI obstruction is mostly associated to abdominal distention and pain and the absence of stool and/or gas output [107].

The management of this condition can vary according to the extent of the obstruction and is mostly pharmacological and/or surgical. 

Nausea and vomiting can be managed through the administration of medications that lower GI secretions (e.g., anticholinergics and somatostatin analogues) and/or anti-emetic agents (i.e., metoclopramide for incomplete bowel obstruction). If medications are not enough to reduce the frequency of vomiting, the positioning of a nasogastric tube or the execution of a gastrostomy represent the most feasible options to decompress the obstruction [108]. Also, in the most severe cases of bowel obstruction, fasting is recommended. Therefore, intravenous hydration and total parenteral nutrition should be offered to these patients to avoid dehydration and electrolyte imbalances, provide adequate caloric intake and avoid the occurrence of malnutrition [108]. 

Opioids are generally sufficient in controlling abdominal pain in these patients [109] although they can worsen their intestinal motility.

Surgery is generally not performed to relieve a lower GI obstruction, and it is reserved to emergency situations (such as strangulation). Recently, however, metallic stent positioning has emerged as an option for treating gastric, small and large bowel obstructions [108]. 

For individuals experiencing sub-occlusion, the following nutritional strategies can be helpful: consuming smaller meal portions that are easier on the digestive system and may help reduce discomfort; opting for a diet lower in fiber (by selecting refined grains over whole grains and avoiding foods with high fiber content), as high-fiber foods can be more challenging to digest and may exacerbate discomfort; removing or avoiding the skins of fruits and vegetables to reduce the fiber content and make these foods easier to digest as well as cooking or peeling fruits and vegetables; staying hydrated with clear fluids to maintain regular bowel movements; and opting for foods with softer textures, such as minced or pureed dishes, which are easier on the digestive system, reduce the risk of intestinal discomfort, and alleviate pain or gas [110]. Dietary adjustments should be made in consultation with a healthcare professional to ensure that the individual receives the necessary nutrients while managing their sub-occlusion effectively.

### 2.8. Dysgeusia and Dysosmia

Around 50–60% of patients undergoing oncologic therapies are expected to have some form of taste and smell alteration [40]. 

Quantitative taste and smell disorders encompass conditions such as ageusia, hypogeusia, anosmia and hyposmia characterized by the complete absence or reduced perception of taste and smell, respectively [111]. On the other hand, dysgeusia and dysosmia are the most common quality alterations [112]. 

In cancer patients, these abnormalities depend on the tumor site (e.g., H&N cancer or trigeminal lesions) [113], nutritional deficiency (vitamin A, B6, B12, zinc and iron deficit) and the treatment used (chemotherapy, radiotherapy, or surgery) and their duration [40]. 

It is well known that platin- and taxane-based chemotherapy can alter taste and smell perception due to neurotoxic damage in cell receptors [40,114]. Additionally, oral and nasal disorders such as mucositis, xerostomia, rhinitis or infections (i.e., SARS-CoV-2) may contribute to the alteration of these pleasant senses.

Although there is no effective treatment, different trials have been conducted over the years [115,116].

Among these, it is worth noting those based on zinc and glutamine that failed to show the prevention of taste alteration. In fact, it has been shown that, if zinc is deficient, its replacement can mitigate and eventually improve gustatory function [19,115]. In severe forms, dysgeusia can be treated with benzydamine or lidocaine oral gel, relieving pain. 

During cancer therapy, it is commonly observed that patients develop heightened sensitivity to bitter and salty tastes while experiencing a decreased perception of sweet tastes, with sour tastes being affected to a lesser extent [19,40]. Many cancer patients also report experiencing a continuous aftertaste that masks the true flavor of food. In response to these challenges, several measures can be taken to enhance the dining experience. To combat the aftertaste and keep the mouth fresh, cancer patients can use alcohol-free mouthwash or menthol products [19]. Maintaining proper oral hygiene is also essential in this context.

To overcome food aversions and improve the intensity of flavor and smell, it is recommended to prepare toppings or dressings that make the food more appetizing [40]. 

Warm meals are generally not recommended for individuals with altered taste perceptions. Instead, opt for cold or lukewarm dishes [117]. Seasoning meat or fish with herbs and spices like rosemary, bay leaf, parsley, thyme, or Jamaica pepper can enhance flavor [40].

Olive oil, salt, and lemon dressings can be used for salads or roasted vegetables to improve taste and palatability. Marinating meat or fish before cooking, or grilling them with acidic products like lemon juice, wine, or beer, can be an excellent strategy to overcome food aversions and enhance flavor [19]. If there is an aversion to red meat, consider trying braised beef, or explore other protein sources like eggs, chicken, or ham. Using plastic cutlery can be beneficial in reducing the metallic taste that some cancer patients experience. Additionally, avoiding eating in overly warm environments can help improve the dining experience. These strategies can help cancer patients maintain their nutritional intake and enjoy their meals despite changes in taste perception and aftertaste. However, it is essential for patients to discuss dietary modifications and preferences with healthcare professionals to ensure they receive adequate nutrition during cancer therapy. 

### 2.9. Anorexia

Among cancer patients, anorexia is the most prevalent eating disorder causing malnutrition, due to a decrease in the intake of nutritive elements, with a subsequent weight loss and ultimately cachexia development [40]. 

It typically occurs in about 50% of all cancer patients and is more common among GI cancers [40,118]. The resulting weight loss and metabolic alterations are considered poor prognostic factors and negative predictors of the response to antineoplastic agents. 

Given the multifactorial pathogenesis, a combination of tumor-related and drugs-related causes have been reported [119]. Surgery, taste and smell alterations and anatomic or functional defects may contribute to a reduced daily caloric intake [40,120]. On the other hand, the side effects of oncology treatments such as mucositis, xerostomia or mouth sores may also induce GI discomfort, reducing appetite and promoting an early satiety [40].

Under normal conditions, the appetite regulatory center is the hypothalamus that controls homeostatic functions through the interaction of multiple signals [40,121]. 

Leptin and ghrelin are hormones that respectively regulate feelings of satiety and hunger. The former functions as a suppressor, while the latter acts as a stimulator of appetite. However, individuals with oncological diseases often experience impaired peripheral signaling, leading to an insufficient response to their energy requirements [40]. 

To tackle implications in terms of hospitalization and mortality, different therapies have been successful in improving appetite, playfulness, and general conditions. The important role of megestrol acetate, which is capable of reducing cytokines production leading to weight gain, has also been pointed out [122,123]. 

Other orexigenic agents are corticoids, progestogens and 5-hydroxytrypamine antagonist (olanzapine) which have proved their effectiveness in increasing fat mass and psychological aspects of cancer patients [124,125]. 

While drug therapy can be a component of treating symptoms of anorexia, it alone is not sufficient. Physical activity should be introduced promptly to help delay weight loss as much as possible [40]. 

However, it is crucial to calculate the patient’s nutritional requirements and address any nutrient deficits that may be present [126,127]. Making food more appealing and tastier can help encourage eating. This can be achieved using toppings, contrasting colors, and serving small portions frequently [40]. Additionally, paying attention to the texture and smell of the food can stimulate oral feeding. Here are some dietary recommendations to address symptoms of anorexia: opt for small, frequent meals, especially well-cooked and energy-rich foods, particularly in the morning; include high-calorie and high-protein foods in the diet (nuts, eggs, cheese, whole milk, oil, butter, and legumes); making homemade smoothies can be an effective way to increase calorie and nutrient intake; juices and cream soups can be easier to consume and provide valuable nutrients; and if red or processed meats are not well tolerated, they should be avoided [40].

But, if despite all this, patients continue losing weight, it is necessary to resort to nutritional supplements or even to artificial nutrition. For all these reasons, family support is becoming increasingly important.

### 2.10. Cachexia

Cancer cachexia (CC) is a complex medical condition often occurring in 50–80% of patients with end-stage disease as a result of metabolic alterations of the tumor and oncological treatment side effects [40]. CC is known for a progressive loss of muscle mass as part of a chronic inflammatory response causing emaciation, metabolic disorders, and physical deterioration [40,128]. 

It is a set of symptoms (anorexia, fatigue), signs (the loss of both muscle and fat mass) and biological alterations (hypoalbuminemia, hyperlacticaemia) that lead to a poor QoL and an increased morbidity and mortality [40].

Therefore, initial approaches should include increasing caloric intake and protein consumption. It could be an option to alleviate malnutrition with oral or intravenous nutritional supplements (enteral or parenteral nutrition) preventing dietary deficiency and stimulating protein anabolism [129,130]. At the same time, it can be useful to resort to appetite-stimulating medications (i.e., corticoids, megestrol acetate, and antidepressants), prokinetics (metoclopramide) and eicosapentaenoic acid (EPA) that might reduce lipolysis and proteolysis [40,131,132]. 

Given the substantial influence of nutritional status, it is advisable to endorse the consumption of high-energy and high-protein foods, particularly in the morning, aiming for a daily intake of 1.5 g of protein per kilogram of body weight, coupled with a caloric intake of 25 to 30 kcal per kg of BW. These foods may include avocados, pudding, cooked cereals, and oats as well as olive oil or butter. Meals should have delicate aromas and flavors, a homogeneous and creamy texture, and a soft consistency. Boiled, cooked, or grilled foods should be opted for as well. Other important suggestions include adding fruit jams and honey to yogurt or salads and pairing aged cheese with soup. Incorporating beef, chicken, or eggs into rice or sandwiches is also advisable [40]. 

Hence, optimal treatment decisions necessitate the involvement of a multidisciplinary team for the early diagnosis and management of this debilitating condition. This collaborative effort should engage family members, physicians, and psychologists to ensure a seamless continuum of care and alleviate the suffering of the patients.

### 2.11. Tailored Recipes

In the field of oncology, dietary modifications should be tailored to specific clinical scenarios. However, preserving the sensory enjoyment of meals and the pleasure of eating poses a notable challenge for cancer patients, whether they are in a hospital setting or at home.

Firstly, patients and their caregivers often lack knowledge about how to process and combine ingredients, since there is a shortage of recipes designed for the oncology context. 

Secondly, many “medical recipes” often prioritize balance but neglect the hedonic component, resulting in nutritious but unappetizing meals. 

In essence, it is essential for patients to receive guidance not only about “what to eat”, but also about “how to prepare it”. The field of gastronomy can provide strategies to enhance not just the quantity but also the quality of food intake. By improving the presentation of meals, food consumption can increase [133]. This way, each meal may transform into a moment of pleasure, contributing to both overall wellbeing and an improved nutritional status. 

**Figure 2:**
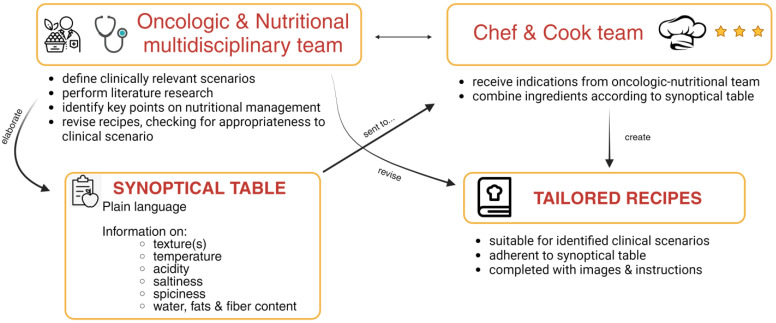
The workflow for the conceptualized multidisciplinary platform to create and clinically validate recipes for clinical scenarios. Made with Biorender.

In order to address this clinical need, we created a workflow consisting of 5 steps (Figure 2).

**Figure 3:**
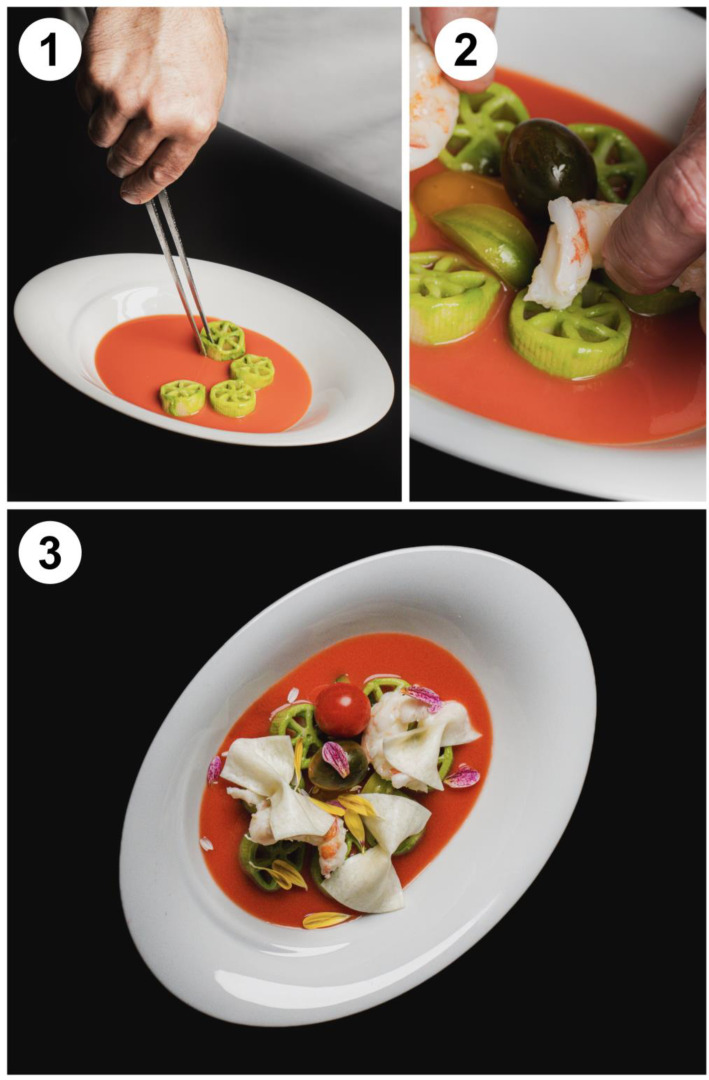
Iconographic material of the recipe for patients and their caregivers.

- First, the oncologic and nutritional teams defined clinically relevant scenarios often encountered in the oncology setting;
- Second, the oncologic and nutritional teams performed a literature research for publications about nutritional management in that clinical scenario;
- Third, a synoptical table (Table 1) to translate in plain language the crucial features of the nutritional approach for that clinical scenario was created;
- Fourth, a chef and his team used that table to create a tailored recipe;
- Fifth, these recipes were analyzed and revised by nutritional team in order to check their appropriateness for clinical scenarios;
- Sixth, the recipes were modified accordingly to the revisions;
- Seventh, accepted recipes were implemented with a figure and detailed how-to instructions (Figure 3, Table 2, Supplementary File S1 for two additional recipes).

This workflow represents a useful and replicable system to create valuable and pragmatic recipes and/or lifestyle interventions for patients with cancer and their caregiver.

### 2.12. Implications and Future Perspectives

Over the centuries, there has been a shift from perceiving nutrition as a means of sustenance and a tool for socialization and hedonism to a pivotal resource for individual and public health. The significant challenge in the field of oncological nutrition in the coming years will be to achieve a higher level of personalization that considers the cultural, social, clinical, biological, genetic, and comorbidity context of the subject [134,135]. This goal is also based on the assumption that tumors exhibit strong heterogeneity, and each of them may be influenced by certain risk factors, such as overweight, exposure to toxins, genetic background, or even parameters once considered clinically neutral, such as the ABO blood group [136,137,138,139].

Although potentially applicable to the field of prevention, our work has focused on the management of cancer patients who have already developed symptoms related to the disease or its treatments. We aimed to establish a pragmatic workflow based on three steps: a literature review for the nutritional management of a specific symptom, the development of recipes adhering to literature recommendations, and a multidisciplinary review of the same.

The advantage of such an approach lies in its flexibility and easy applicability to almost all nutritional scenarios, not limited to oncology. However, the major limitation of this work is the need to validate, through a clinical study, whether this operational model is indeed effective in improving the quality of life of cancer patients. In addition, although we have not performed a systematic review on each oncologic symptom, it is already evident that there is a paucity of literature on these topics. 

## 3. Conclusions

Customized nutrition in oncology continues to be a challenge due to the limited scientific data and the availability of suitable recipes. In this context, we have introduced a highly practical multidisciplinary framework for tailoring food preparation to address various common clinical scenarios encountered in oncology patients. Enhancing the quality of life holds a central position in contemporary medicine, especially in the field of oncology, and personalized recipes have the potential to be a strategic resource for improving nutritional wellbeing and bringing happiness to the numerous patients who must cope with cancer.

Preserving the hedonic value of meals and the enjoyment of eating is a significant challenge when it comes to cancer patients. However, gastronomy offers strategies to enhance not just the quantity, but also the quality of food consumption. Elevating the presentation of meals can lead to increased food intake. Consequently, each meal transforms into a moment of pleasure, contributing to both overall wellbeing and an enhanced nutritional status.

## Figures and Tables

**Table 1:** The synoptical table to translate in plain language the crucial features of nutritional approach for each clinical scenario.

	Xerostomia	Stomatitis	Diarrhea	Nausea/Vomiting	Dysphagia (Mechanical)	Dysphagia (Neurological)	Sub-Occlusion	Dysgeusia and Dysosmia	Anorexia	Cachexia
Texture (IDDSI scale)	0–5	0–4	Any	Any	0–4	3–5	0–4	Any	Any	Any
Multiple textures	Any	Any	Any	Any	Avoid	Any	Any	Any	Any	Any
Temperature	Any	Low	Normal	Low	Any	Any	Any	Any to warm/hot	Any	Any
Sourness	Mild to high	Very low	Normal to low	Low	Any	Any	Any	Any	Any	Any
Sapidity	Any	Low to very low	Normal to low	Normal to low	Any	Any	Normal	Normal to very low	Any	Any
Spiciness	Any	Avoid	Low	Low	Any	Any	Normal to low	Normal to very high	Normal to very high	Normal to very high
Hydration	High	High	Low	Normal	High	High	High	Any	Any	Any
Fattiness	Normal	Normal	Low	Low to very low	High to very high	Normal	Any	High	High to very high	High to very high
Amount of fiber	Any	Any	Low to very low	Low	Avoid	Normal to avoid	Avoid	Any	Normal to low	Normal to low
Leading nutritional points	Proper oral hygiene Introducing different flavors Avoiding sour foods Acupuncture during and after radiation for H&N	Saline and sodium bicarbonate mouthwashes Oral cryotherapy Preferring soft or semisolid, mild-textured foods Opting for cool or room-temperature foods	Abundant oral liquids with solutions rich in electrolytes and glucose Probiotics supplementation Small and fractioned meals rich in soluble fibers Avoiding foods and beverages with high osmolarity, caffeine and theine	Eating small and frequent meals Having light snacks between meals Introducing ginger, chamomile, peppermint and cardamom Reducing liquid intake during meals Avoiding spicy, fatty, salty food and strong flavors Preferring bland foods, soft fruits or vegetables	Preferring more liquid forms of foods Reducing fiber content	Consuming small, fractionated semi-solid meals and thick liquids Prioritizing hard texture and fatty food Avoiding sticky or bulky foods, mixed textures	Consuming smaller meal portions Opting for a diet lower in fiber and softer-textured foods Selecting refined grains Avoiding foods with high fiber content, skins of fruits and vegetables	Preparing toppings or dressings Marinating meat or fish before cooking, or grilling them with acid products Opting for cold or lukewarm dishes Seasoning meat or fish with herbs and spices like rosemary, bay leaf, parsley, thyme, or Jamaica pepper Avoiding warm meals	Using toppings, contrasting colors, and serving small portions Opting for small, frequent meals, especially well-cooked and energy-rich foods, particularly in the morning Including high-calorie and high-protein foods in the diet (homemade smoothies, juices and cream soups)	Endorsing high-energy and high-protein foods with delicate aromas and flavors Preferring a homogeneous and creamy texture and a soft consistency Protein 1.5 g/day per kilogram of BW Opting for boiled, cooked, or grilled Adding fruit jams and honey to yogurt or salads and pairing aged cheese with soup

**Table 2:** Example of a recipe for cachexia/anorexia with its nutritional profile.

**Fiori sulle farfalle (Flowers on butterflies)** *Preparation and servings for two people* **For the tomato soup:** Ingredients: Red grape tomatoes 150 g Red cherry tomatoes 150 g Extra virgin olive oil 15 g Salt 3 g Dulcita brown sugar 0.5 g Procedure: Blend together date and cherry tomatoes, then stir in the remaining ingredients. Strain through a sieve. **For the cold pesto pasta:** Ingredients: *Benedetto Cavalieri* pasta wheels 60 g Basil pesto 30 g Water 10 g Procedure: Cook the pasta wheels in boiling salted water (cooking time: 12–13 min), then cool them quickly in cold water and dry them using a paper towel. Toss them with a drizzle of extra virgin olive oil and basil pesto diluted with a spoonful of warm water. **For the mozzarella butterflies:** Ingredients: Cow’s milk mozzarella cheese 60 g Procedure: Cut the mozzarella into chunks and heat it in a microwave oven, with a little of its preserving water, until it becomes stringy and malleable. Drain off the excess liquid, place it between two sheets of baking paper and stretch it using a rolling pin (reaching a 2 mm thickness). Cut some 3 × 6 cm rectangles; pinch them in the center by means of the tip of a pair of tongs (previously heated in boiling water) to make butterflies **For the steamed langoustines:** Ingredients: Langoustines 6 pieces Procedure: Peel and clean the langoustines. Steam for about 1 min. Cut them into medallions and season them with salt and a drizzle of extra virgin olive oil. **Plating** Pour the cold tomato soup at the base of a soup bowl. Form a circle with the basil pesto pasta wheels, put various colored cherry tomatoes in the middle and season them with extra virgin olive oil and salt. Lay the mozzarella butterflies on top of the pasta, arrange the langoustines medallions and finish the dish with edible flowers’ petals. **Caloric and nutrients labels (for one serving)**	
Calories, Kcal	484
Proteins, g	21
Fats, g	31
Saturated fatty acids, g	8
Monounsaturated fatty acids, g	18
Polyunsaturated fatty acids, g	3
Carbohydrates, g	31
Sugars, g	7
Fiber, g	3

## Data Availability

Not applicable.

## Supplementary Materials

The following supporting information can be downloaded at https://www.mdpi.com/article/10.3390/nu15245068/s1, File S1: supplementary recipes.

## Abbreviations

5-fluorouracil, 5-FU; cancer cachexia, CC; chemotherapy, CINV; radiation therapy, RINV; eicosapentaenoic acid, EPA; gastrointestinal, GI; head and neck, H&N; percutaneous endoscopic gastrostomy, PEG; nausea and vomiting, N/V; neurokinin 1, NK1; quality of life, QoL.
