# Supplementary material for: Nutritional Management of Oncological Symptoms: A Comprehensive Review
Source: Nutrients. 2023 Dec 11;15(24):5068. doi: 10.3390/nu15245068 (PMC10745914; doi:10.3390/nu15245068)

Supplementary Materials

Luce Fluida (sunshine in a plate) (mechanic dysphagia)

Preparation and servings for two people

For the mango and yellow tomato soup:

Ingredients:

- Ripe mango 60 g
- Yellow grape tomatoes 150 g
- Water 20 g
- Salt 1 g
- Extra virgin olive oil 15 g

Procedure:

- Blend the yellow grape tomatoes together with cold water and the ripe mango.
- Pass the mixture through a fine sieve and emulsify it with salt and extra virgin olive oil using an immersion blender.

For the yellow tomato mousse:

Ingredients:

- Yellow grape tomatoes 180 g
- Unsweetened plain soy milk 50 g
- Salt 1 g
- Dulcita brown sugar 0.5 g

Procedure:

- Blend and sieve the yellow grape tomatoes to obtain a puree.
- Emulsify the puree with the soy milk, salt and brown sugar, by using an immersion blender.
- Add tomato emulsion to a siphon and load with a charge for cream. Refrigerate for at least 4 h.

For the beet mousse:

Ingredients:

- Fresh beets 300 g
- Red grape tomatoes 150 g
- Salt 1 g
- Dulcita brown sugar 0.5 g

Procedure:

- Wash the beets and centrifuge them to obtain a juice
- Blend and sieve the red grape tomatoes and combine with the beet juice
- Blend the mixture with salt and brown sugar
- Add beet mixture to a siphon and load with a charge for cream. Refrigerate for at least 4 h

For the pistachio and basil sauce:

Ingredients:

- Pistachios, unshelled 100 g
- Water 150 g
- Salt 1 gr
- Extra virgin olive oil 50 g
- Cold-pressed corn oil 150 g
- Fresh basil 15 g
- Lemon juice 3 g

Procedure:

- Blanch the pistachios in boiling water for 3 min then drain and let cool.
- Peel pistachios and blend with water, salt and extra virgin olive oil.
- Once smooth, add the lemon juice.

For the red shrimp cream:

Ingredients:

- Red shrimp 150 g
- Unsweetened plain soy milk 50 g
- Extra virgin olive oil 10 g
- Water 10 g
- Salt 0.5 g

Procedure:

- Clean the shrimp and freeze or blast chill at -20°C for at least 24 h.
- Blend them with the soy milk, water, extra virgin olive oil and salt, until smooth.

For the black olive sauce:

Ingredients:

- Black olive paté 70 g
- Water 40 g

Procedure:

- Blend the black olive paté with water, until emulsified.

Plating:

- Add two spoonfuls of mango and tomato soup to a soup bowl, then add three teaspoons of pistachio sauce spaced out across the bowl, using a spoon or pastry bag. Top with three mounds of red shrimp cream from the siphon. Also decorate with black olive sauce.
- Complete the dish by adding the beet and the tomato mousses and a drizzle of extra virgin olive oil.

**Calories, Kcal**	1618
**Proteins, g**	25
**Fats, g**	154
**Saturated fatty acids, g**	21
**Monoinsaturated fatty acids, g**	77
**Polyinsaturated fatty acids, g**	48
**Charbohydrates, g**	26
**Sugars, g**	24
**Fiber, g**	14

Caloric and macronutrients label (for 1 serving)

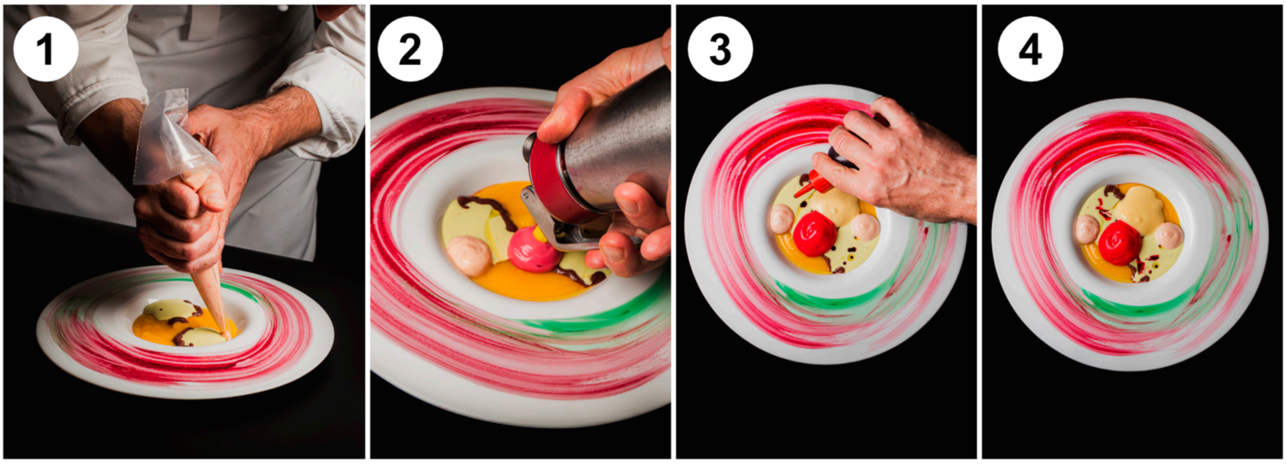

Legumi e legami (bean and scallop soup) (constipation)

Preparation and servings for two people

For the legume and seaweed soup:

<u>Ingredients:</u>

- Fresh Cannellini beans 150 g
- Fresh Borlotti beans 150 g
- White onion 40 g (half of a small white onion)
- 1 bay leaf
- Fresh broad beans 300 g
- Chopped mixed seaweed (kombu, wakame, sea lettuce, Irish moss) 50 g
- Extra virgin olive oil 50 g
- Salt

<u>Procedure:</u>

Add fresh Cannellini and Borlotti beans to a saucepan together with the onion and bay leaf. Cover with cold water and bring to a simmer.

- **Note:** dried beans can be used, as well, providing they have been soaked until they triple their initial dry weight.
- Once the beans are cooked, blend 3/4 of them with a little cooking water to make a smooth cream (if needed, further dilute it with water or broth) and add salt and extra virgin olive oil to taste. **Note:** to further enrich the flavour, other legumes like blanched fava beans, chick peas, lentils, peas or red soybeans can be added.
- Dress the cream with the chopped mixed seaweed, previously blanched or rehydrated (if dried seaweed is used).

For the scallops:

Ingredients:

- Scallops 4 pieces
- Extra virgin olive oil 15 g
- Umeboshi 10 g
- Ponzu 5 g
- Dried seaweed flakes 3 g
- Salt

Procedure:

- Carefully clean the scallops.
- Add salt and extra virgin olive oil to taste and grill the scallops on both sides.
- Dress them with extra virgin olive oil, umeboshi puree, ponzu and dried seaweed flakes.

For the fried onion or leek:

Ingredients:

- *00* flour 35 g
- Cornstarch 15 g
- Sparkling water 45 g
- Ice cubes
- Onion/leek rings 30 g

Procedure:

- Make a light tempura with sparkling water, ice cubes, *00* flour and cornstarch.
  Note: The tempura batter should be kept very cold, ice cubes could also be placed at the bottom of a bowl in contact with the tempura.
- Dip the onion/leek rings in the tempura and fry them in olive oil at 175°C.
- Drain the rings, using paper towels to absorb the excess fat and add a small pinch of salt.

Plating:

- Pour two ladles of the legume and seaweed soup in a bowl and mix it with the remaining legumes (kept as a whole).
- Place two grilled scallops on top of the soup.
- Complete the dish with the dried seaweed flakes, fried onion/leek rings and a drizzle of extra virgin olive oil.

**Calories, Kcal**	694
**Proteins, g**	26

**Fats, g**	34
**Saturated fatty acids, g**	5
**Monoinsaturated fatty acids, g**	24
**Polyinsaturated fatty acids, g**	3
**Charbohydrates, g**	60
**Sugars, g**	6
**Fiber, g**	29

Caloric and macronutrients label (for 1 serving)

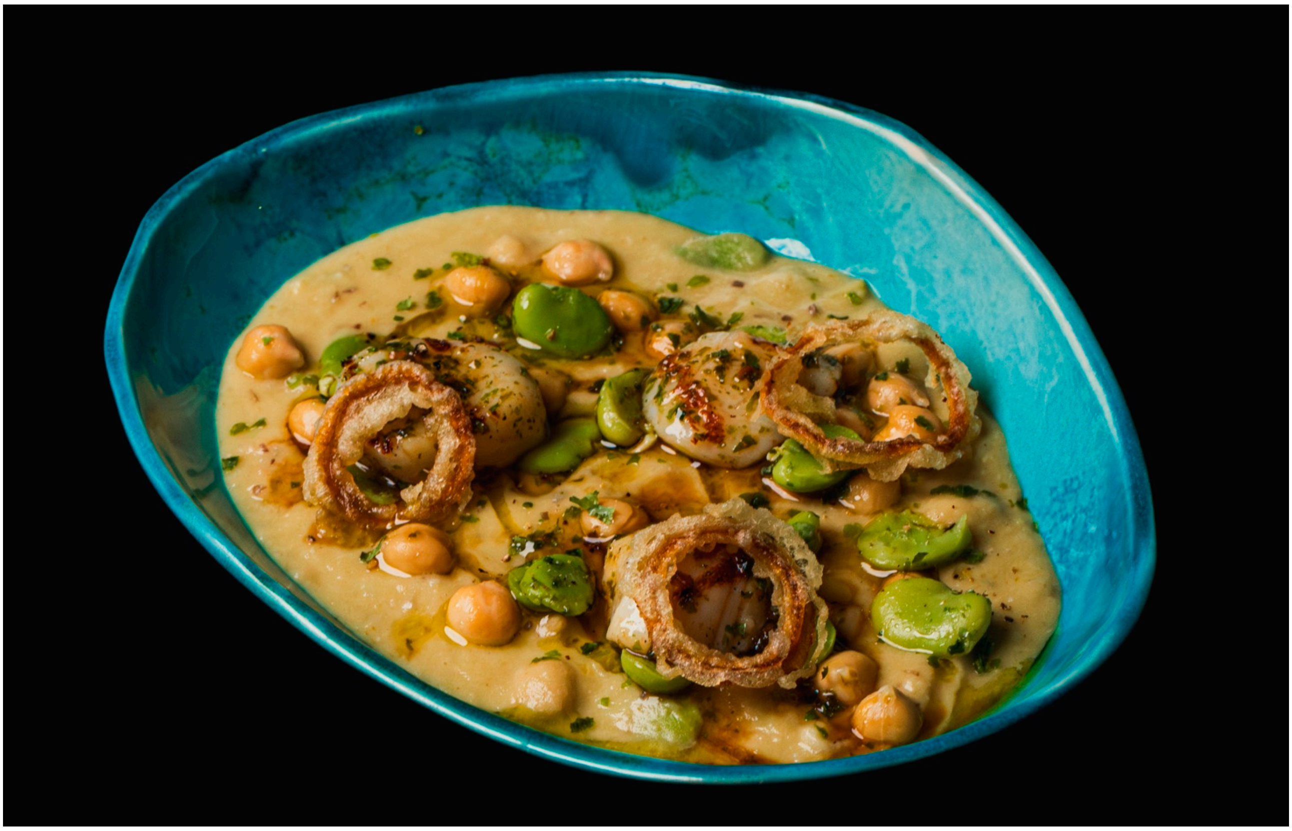

Supplement: Supplementary file 1 [file nutrients-15-05068-s001.zip › nutrients-2723204-supplementary.pdf]
